# Comparison of Two Methods for the Determination of Selected Pesticides in Honey and Honeybee Samples

**DOI:** 10.3390/molecules23102582

**Published:** 2018-10-09

**Authors:** Żaneta Bargańska, Piotr Konieczka, Jacek Namieśnik

**Affiliations:** Department of Analytical Chemistry, Faculty of Chemistry, Gdańsk University of Technology (GUT), Narutowicza 11/12 street, 80-233 Gdańsk, Poland; piotr.konieczka@pg.edu.pl (P.K.); jacek.namiesnik@pg.edu.pl (J.N.)

**Keywords:** honey, honeybees, pesticides, LC-MS/MS, GC-MS/MS, QuEChERS, environmental monitoring, Eco-Scale

## Abstract

Developed and validated analytical methods for the determination of a wide spectrum of pesticide residues in honey and honeybee samples after the modification of QuEChERS extraction in combination with gas chromatography–tandem quadrupole mass spectrometry (GC-MS/MS) and liquid chromatography–tandem quadrupole mass spectrometry (LC-MS/MS) were discussed and compared. The developed methods were evaluated regarding the utilized equipment and reagents using Eco-Scale and compared in terms of extraction time, accuracy, precision, sensitivity and versatility, with similar procedures. The results proved that the QuEChERS protocol in combination with LC and GC techniques fulfills the requirements of green analytical chemistry, so it can be used as a tool in environmental monitoring. The recovery was 85–116% for honey and 85.5–103.5% for honeybee samples. The developed methods were successfully applied in monitoring real samples collected from three districts of Pomerania in Poland. Analysis of real samples revealed the presence of the following pesticides: bifenthrin, fenpyroximate, methidathione, spinosad, thiamethoxam, triazophos, metconazole and cypermethrin at levels higher than the MRLs established by the EU.

## 1. Introduction

Plant protection products (PPPs) are widely used in agriculture to protect crops. Due to their different chemical structures, pesticides belong to different classes and chemical groups (e.g., fungicides, insecticides, growth regulators etc.). Therefore, the development of a universal and green analytical method for the determination of a wide range of pesticide residues in one analytical cycle, poses a challenge for scientists.

Honey is produced by honeybees from nectar, pollen or honeydew, which they collect from different plants [1]. Honey contains about 300 substances (e.g., carbohydrates, vitamins, enzymes, etc.), so it has healing properties and is the source of many trace minerals necessary for human health. The presence of individual compounds (belonging to several groups of chemical compounds) and their amount (concentration) depends on the type of plant, apiary location and beekeeper practices [2]. For these reasons, honey is considered as a valuable food product. The presence of xenobiotics in honey or other bee products may deteriorate their quality and properties [3,4,5]. Therefore, it is necessary to control the level of harmful substances to human and honeybees’ health in this specific type of food product.

In recent years, an inexplicable and sudden fall of bee colonies has been observed in the apiaries. The phenomenon of mass extinction of honeybees was named by scientists Colony Collapse Disorder (CCD) [6,7,8,9] and has become a serious threat to agriculture, economy, the environment and people. It is due to pollinating insects that plants give abundant crops and the animals grazing on the meadows give nutritious food products.

Honeybees and their products are a typical example of biological material that is characterized by a complex matrix composition. The stage of sample preparation for analysis is a key element in all analytical procedures for the determination of xenobiotics, especially pesticides. Honeybees and honey contain large quantities of interfering substances that may have an adverse effect on the result of the analysis. Therefore, the development of analytical procedures able to process samples in an economic way and, at the same time, fulfilling requirements of green chemistry is crucial.

The popular extraction technique of a wide range of pesticides is the quick, easy, cheap, effective, rugged and safe (QuEChERS) method. In the first stage liquid-liquid extraction (LLE) with salting-out (standard MgSO_4_ and NaCl salts) is carried out, followed by clean-up by dispersive solid phase extraction (dSPE) using primary and secondary amine bonded silica (PSA). This basic method was proposed [10] for extraction of pesticides from food products. However, for several years, the basic QuEChERS procedure has been modified many times and used for determination of pesticide residues in honey [11,12], wax [13,14], pollen [15], honeybees [16,17,18], royal jelly [19,20], bumblebees [21,22] and beebread [23,24,25], coupled with analysis using LC-MS/MS and GC-MS/MS. Modified QuEChERS has also been used in the analysis of perchlorate (ClO^4−^) and bromate (BrO^3−^), biogenic amines, and quaternary ammonium compounds in various kinds of food matrix [26,27].

In summary, it is necessary to develop new analytical methodologies or modify the ones already existing that enable the simultaneous determination of a wide range of compounds from bee and bee product samples in one analytical cycle. Until recently, researchers have been developing methods for determination only a few pesticides (from the same classes and groups of chemicals) using dedicated methods and selective detectors [28]. Nowadays, the most convenient detector for the analysis of pesticide residues is a high resolution mass spectrometer, coupled with gas or liquid chromatography. The choice of instrumentation is mostly dependent on the type and class of pesticides. The use of the mass analyzer makes it possible to meet the requirements of the sensitivity and selectivity criteria of the analytical method [29]. Liquid chromatography-tandem mass spectrometry (LC-MS/MS) and gas chromatography-tandem mass spectrometry (GC-MS/MS) are often used for analysis of veterinary drug and pesticide residues in bee and bee product samples [30,31,32].

The concept “green chemistry” is constantly being developed and has been since the 1990s. The introduction of the concept of sustainable development into the analytical laboratory has been called “green analytical chemistry”. The parameters that determine the green nature of chemical analysis include: elimination (or at least limitation) of the use of chemical reagents (especially organic solvents) or replacing them with less toxic substances; reduction of emissions of solid waste and sewage generated in the analytical laboratory and reduction of work and energy consumption in analytical procedures (calculated per one analyte). There are few tools to evaluate analytical procedures in terms of meeting the requirements of green analytical chemistry. Eco-Scale is one such tool [33,34,35].

In this study we developed and validated analytical methods for the determination of selected pesticides in honey and honeybee samples after the modification of QuEChERS extraction in combination with gas chromatography–tandem mass spectrometry (GC-MS/MS) and liquid chromatography-tandem mass spectrometry (LC-MS/MS) and assessed them using the Eco-Scale tool. The target pesticides were chosen on the basis of information from beekeepers that are members of the Regional Beekeepers Association in Gdansk (Poland) in cooperation with farmers, based on anonymous surveys and results obtained in the previous studies. The methods were evaluated according to the SANCO guidance: Method Validation and Quality Control Procedures for Pesticide Residue Analysis in Food and Feed [36] for their repeatability, linearity, recovery, limit of detection and quantification. The validated methods were applied for determination of pesticide residues in real samples from three counties of Pomerania (Poland). The proposed analytical scheme offers: (i) simplicity, low operation cost, high extraction efficiency and enrichment at a relative short extraction time (~5 min); (ii) satisfactory metrological parameters (e.g., LOD, LOQ, recovery), (iii) sample preparation without the necessity to evaporate the solvent and reconstitute it prior to analysis; (iv) determination of pesticide residues on the level of ng/g, allowed by the use of the proposed methods, and (v) small sample volume. Application of presented methods in routine analyses would allow a significant reduction of reagents, sample and time. The developed analytical procedures make it possible to monitor pesticides belonging to various classes and can be used in environmental monitoring.

## 2. Results and Discussion 

### 2.1. Extraction Procedure

Honeybees always contain large amount of beeswax and proteins. Therefore, their analysis is difficult. The basic QuEChERS method does not eliminate the wax from bee organisms. Therefore, addition of a small amount of *n*-hexane eliminates such an interference. 

**Figure 1:**
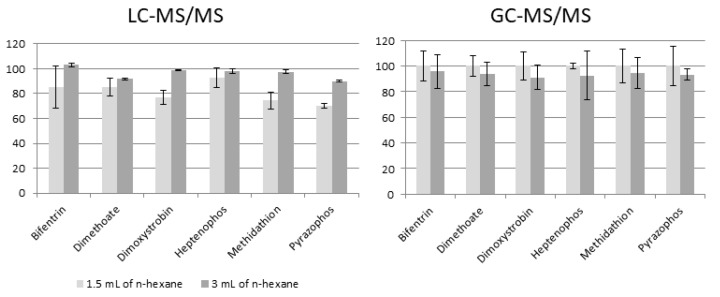
Differences in recovery found during the extraction process when using 3 mL versus 1.5 mL of n-hexane for removing wax from honeybee samples using modified QuEChERS method for selected pesticides.

In previous studies we added 3 mL of *n*-hexane in QuEChERS extraction, now the proposed amount is 1.5 mL. Figure 1 presents differences in recovery found during the extraction process when using 3 mL versus 1.5 mL of *n*-hexane for removing wax from honeybee samples using modified QuEChERS method for selected pesticides.

Addition of 1.5 mL of *n*-hexane in the extraction step, compared to the addition of 3 mL of this organic solvent, caused a slight increase in analyte recovery with a comparable residual standard deviation (except for heptenophos and pyrazophos)—for GC and caused a slight decrease in analyte recovery—for LC. The residual standard deviation was slightly higher than before QuEChERS modification, but not more than 20%.

### 2.2. Validation Study

#### 2.2.1. Linearity and Matrix Effects

The three and five-point-calibration curves in solvent and in the two matrices (honey and honeybees) were constructed and compared at the LOQ, 3LOQ, 6 LOQ ng/mL and LOQ, 25, 50, 75, 100 ng/mL concentration levels with the addition of 100 ng/g TPP as internal standard for LC and GC analysis, respectively. The results of the present study showed that 68% of the pesticides presented correlation coefficients (R2) higher than 0.997, and 20% of the compounds were lower than 0.995 in all calibration curves. These results are included in Table S2 (Supplementary Data).

It is obvious that the matrix effects have a different character when using liquid chromatography and gas chromatography [37]. However, some substances that do not interfere with the analysis of pesticide residues in biological samples may accumulate in the system (especially in GC-MS/MS) and cause problems related to the resistance of the analytical method, such as the amplification signal, or the opposite suppression of some susceptible pesticides [38].

**Figure 2:**
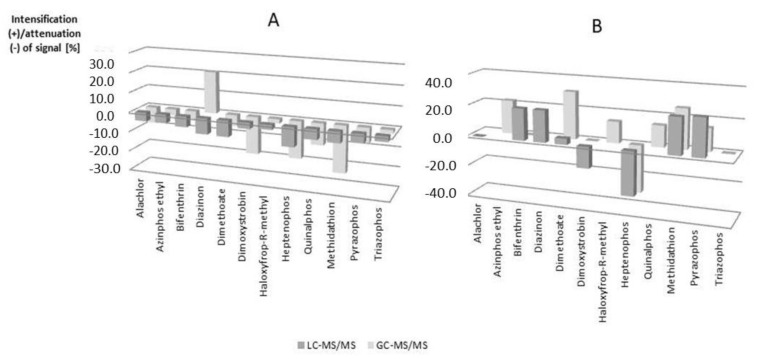
Distribution of matrix effects for selected pesticides: **A** Honey **B** Honeybee.

Depending on the analyzed samples, different matrix effects (ME) could be observed. In most cases, the impact of the matrix was in the range from −20% to +20% and was considered as a mild signal suppression or enhancement effect. If ME was higher than ±20%, it was considered as a medium effect. Such a distribution can depend not only on the matrix effect but also on the compound-matrix combination, and finally the detection technique (Figure 2).

From the data presented in Figure 2, it can be concluded that the matrix effect for honeybee samples was higher than for honey samples when GC-MS/MS analysis was used. Only for heptenophos the ME was similar in LC and GC analysis. For honey samples ME in most cases the signal was weakened. Only for diazinon the signal was strengthened in GC analysis.

#### 2.2.2. Limit of Detection and Recovery Study

The limit of detection (LOD) was calculated using the equation LOD = 3.3 × SD/b, where b is the slope of the calibration curve and SD is the residual standard deviation of this curve. The limit of quantification was calculated as LOQ = 3 × LOD. 

**Figure 3:**
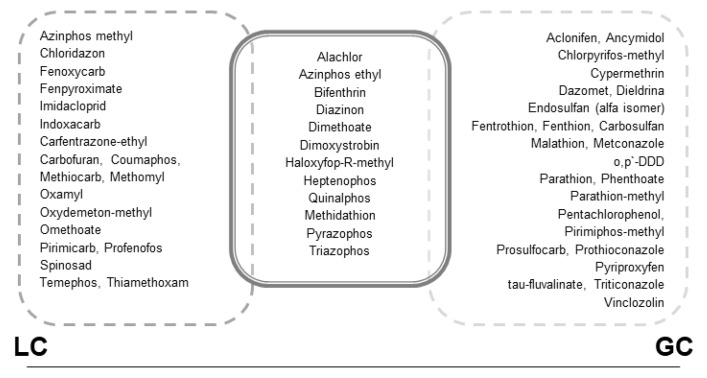
Information on the identification and detection of the selected analytes using the developed analytical methods (LC and GC).

Recovery of the analytes and the method repeatability studies were carried out at three levels of fortification (3LOD, 20 ng/g and 50 ng/g) for GC analysis and two levels of fortification (3LOD, LOQ) for LC analysis by adding known quantities of pesticides standard solution to aliquots of 0.5 g of homogenized honey and honeybee samples, each in five replicates. Extracted “blank” matrices may have contained some of the investigated pesticides. Therefore, blank-correction from the calibration samples and also from the spiked samples was necessary during the analysis. The recoveries calculated using the matrix-matched calibration curves were in the range of 85–116% for honeybee samples and 85.5–103.5% for honey samples, which meets the recommendation of SANCO guideline. The analytical parameters of the proposed QuEChERS methods in spiked honey and honeybee samples are listed in Table S3 (Supplementary Data). Figure 3 presents information on the ability to identify and detect the selected pesticide residues using the developed analytical methods.

### 2.3. Uncertainty Budget

Expanded uncertainty was calculated based on the formula (1) and was presented in Table 1 for selected pesticides. Uncertainty budget reflects all the sources of uncertainty which have an impact on the final results of pesticide residues analysis:

(1) $$U_{x}=k\sqrt{u_{precision}^{2}+u_{cal}^{2}+u_{recovery}^{2}}$$

where: *U_x_*—expanded relative uncertainty, *k*—coverage factor (for probability 95%, *k* = 2), *u_precision_*—standard relative uncertainty arising from precision, *u_cal_*—standard relative uncertainty arising from calibration, *u_recovery_*—standard relative uncertainty arising from trueness (as recovery).

### 2.4. Eco-Scale Calculation

In this article we used a comprehensive tool for semi-quantitative evaluation of analytical methodologies, named Eco-Scale [39,40]. This approach assumed that the ideal green analysis has a value of 100. The sum of penalty points for the whole procedure should be included in the Eco-Scale calculation, according to the following formula:

Analytical Eco-Scale = 100 − total penalty points(2)

The result of calculation is ranked on a scale, where the score >75 represents excellent green analysis, >50 represents acceptable green analysis, and <50 represents inadequate green analysis.

The penalty points for the proposed methods for determination of pesticide residues in honey and honeybee samples using GC-MS/MS and LC-MS/MS are given in Table 2 and Table 3, respectively. Penalty points include several parameters, such as extraction time, use of organic solvents in analytical methodology or laboratory equipment. Thanks to this assessment, suitable substrates or solvents can be selected, which not only meet the requirements of green analytical chemistry, but also may be inexpensive in exploitation. Although, thanks to the use of Eco-Scale we only get a grade—a numerical result, that solution allows us to improve certain stages in the analytical framework, in terms of environmental impact, when developing analyte determination procedures.

The developed analytical methods meet the requirements of green analytical chemistry. Determination of pesticide residues in honey and honeybee samples using the proposed method—GC-MS/MS, represents excellent green analysis. Its high rank in the Eco-Scale (grades 90 and 82, respectively) results from the minimal use of chemical reagents and organic solvents. However, using LC-MS/MS for the determination of pesticide residues in honey and honeybee samples represents acceptable green analysis. The Eco-Scale grades (62 and 70, respectively) were lower than 75.

Table 1 and Table 2 also show the penalty points for pesticide residues determination in honey and honeybee samples before and after the modification of QuEChERS extraction. Therefore, changes in the Eco-Scale rating can be compared. Although the changes achieved in the Eco-Scale assessment are small, they indicate an impact of the elimination of organic solvents in the analytical process on the environment. The main disadvantage of the Eco-Scale assessment is too wide a range for calculating the penalty points (PPs): <10 mL (g)—1 PPs; 10–100 mL (g)—2 PPs and for >100 mL (g) —3 PPs. Total penalty points value is a multiplication of the amount PP and hazard PP.

### 2.5. Sample Analysis

The methods developed in this study were applied to determine pesticide residues in 15 honey and honeybee samples from three counties of Pomerania (Poland): Kartuzy, Gdańsk and Tczew. 

Based on the results of sample analysis (see Table S4, Supplementary Data), and also on the previous studies [41,42,43], it can be concluded that pesticide residues accumulate to a large extent in bee organisms, and then in honey. In 22.6%, 18.1% and 10% of the honeybee samples quantifiable amounts of pesticides were determined in Kartuzy, Gdańsk and Tczew counties, respectively. For the same counties, 5.8%, 4.5% and 4.4% of the honey samples exhibited quantifiable amounts of pesticides.

Honeybees produce honey from flower nectar and/or honeydew. The transformation of these substrates into honey takes place in the body of bees with the help of appropriate enzymes secreted from salivary glands (including invertases, amylase or glucose oxidase). So, honey is a product already “processed” by the bee organism. Therefore, the content of impurities in honey is lower than in the bee’s body. However, regardless of penetration of the harmful substance into the body of the insect, it is a fact that the xenobiotic undergoes many processes in this body (including adsorption, distribution in tissues and organs, or biotransformation into individual components and excretions from the body). A small percentage of the toxic substance reaches its target site unchanged, disrupts certain metabolic pathways and connects to receptors of cells susceptible to its influence. Therefore, the pesticide residues cannot always be detected, and the cause of death of the pollinating insect will be unknown despite the usage of the developed and validated analytical procedures.

**Figure 4:**
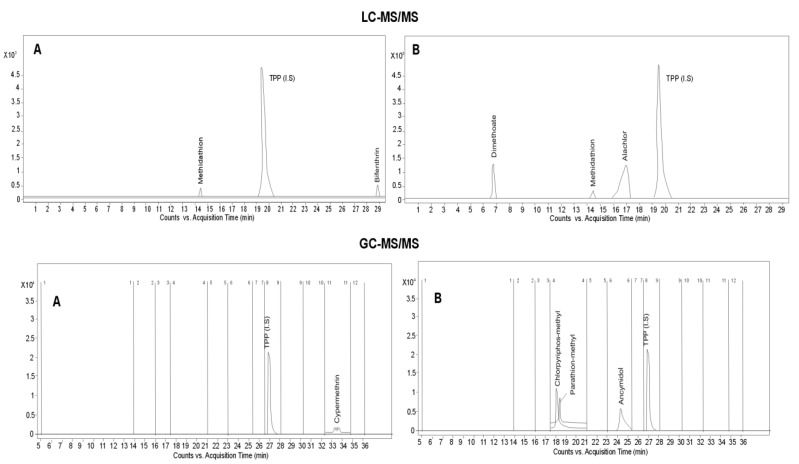
The sample chromatograms obtained during analysis of extracts from real sample in which selected pesticide residues was detected above MRLs level: **A**—honey samples, **B**—honeybee samples.

Example MRM chromatograms obtained during analysis of extracts from honey and honeybee samples in which selected pesticide residues was detected above MRLs level are illustrated in Figure 4.

#### 2.5.1. Honey Samples

- Twenty eight pesticide residues were detected above the LOQ, except for bifenthrin, fenpyroximate, methidathione, spinosad, thiamethoxam and triazophos, as their concentrations were determined at a level higher than the maximum residue levels (MRLs) when using the LC-MS/MS;
- Seven pesticide residues were detected above the LOQ, except for metconazole and cypermethrin, as their concentrations were determined at a level higher than the maximum residue levels (MRLs) when using the GC-MS/MS;
- In 79% of samples the pesticide residues were detected at >LOQ level, in particular from the group of acaricides, herbicides, fungicides, pyrethroids, insecticides and growth regulators.

Honey samples in which pesticide residues have been determined were varieties of multiflorous honey or rape honey. Therefore, it can be concluded that honeybees collected substrates for the production of these honeys during the spraying of plants and crops (e.g., rape, apple, pear, etc.).

#### 2.5.2. Honeybee Samples

- Thirteen pesticide residues were detected above the LOQ, mainly alachlor, dimethoate and methidathion (when using the LC-MS/MS);
- Thirteen pesticide residues were detected above the LOQ, mainly ancymidol, chlorpyrifos-methyl, endosulfan (alfa isomer), haloxyfrop-R-methyl and parathion-methyl and pyrazophos (when using the GC-MS/MS).
- In 67% of samples the pesticide residues were detected at >LOQ level, in particular from the group of insecticides, herbicides, fungicides, pyrethroids and growth regulators.

The obtained results allow to state that the death of honeybees may have occurred as a result of poisoning with pesticides; however not by direct contact, but indirectly, by way of long-term effects or subacute poisoning.

### 2.6. Comparison of the Proposed Methods with Other Methodologies

Many aspects of determination of pesticide residues in honeybees and bee products by GC-MS/MS and LC-MS/MS (Table S5, Supplementary data) have been discussed in the literature. These data confirm that the LC and GC techniques in combination with tandem mass spectrometry do not solve all the problems associated with the determination of pesticide residues. However, both chromatographic techniques are necessary in trace analysis. It can be concluded that the preferred chromatographic technique in conjunction with the MS/MS detector for determination of pesticides residues in honeybee and bee product samples is liquid chromatography. It enables the determination of a wide spectrum of analytes without derivatization. However, mainly due to the use of the mobile phase, the LC technique is less positively assessed using Eco-Scale. 

The main differences between the developed procedures and other published methods for determination of pesticides residues in honey and honeybee samples pertain to the time of the stage of extraction and clean-up. Based on the data presented in Table S5 it can be concluded that the developed methodologies introduce a new trend in the process of determination of a wide range of pesticides in honey and honeybee samples in comparison with other already published methods, because sample preparation time was short. 

The major advantage of the proposed methods is analysis of solvent extracts with small sample mass (0.5 g) and short time of sample preparation (~5 min).

## 3. Materials and Methods

### 3.1. Chemicals and Instrumentation

The chemicals and analytical procedures used for determination of pesticide residues in honey and honeybee samples are shown in Table S1 (Supplementary Data). The stock standard solutions were stored at −18 °C. The calibration standards and working standards were prepared by dilution with acetonitrile on the day of analysis. For the multiple reaction monitoring (MRM) measurements, the collision energies and retention time were described in previous studies [41,42,43].

### 3.2. Sample Collection and Preparation

Fifteen honey and 15 honeybee samples were collected by beekeepers that are members of the Regional Beekeepers Association in Gdansk (Poland) from three counties of Pomerania (Poland): Kartuzy, Gdańsk and Tczew, in the spring/summer of 2012 and 2013. Sample numbers: 1–5 from Gdańsk County, 6–10 from Kartuzy County and 11–15 from Tczew County. The samples were collected at the time when farmers were using the plant protection products (PPPs) around the apiary. Beekeepers noticed negative effects for bee colonies after agrochemical treatments (e.g., dead honeybees, small reproduction). Honey samples were stored at 4 °C in glass containers. Honeybee samples were lyophilized and then ground in an agate mortar and stored at −18 °C.

**Figure 5:**
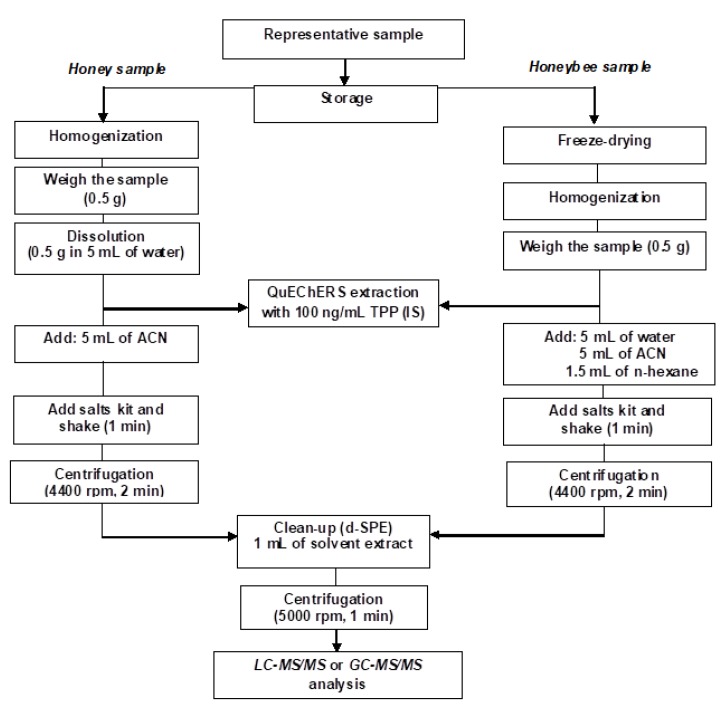
Scheme of pesticide extraction procedures from honey and honeybee samples using the QuEChERS extraction before final analysis.

The sample preparation steps had been described in previous studies [41,42,43] but three changes were made. The first change was reducing the centrifugation time to 2 min, as it was sufficient for complete phase separation, therefore time of extraction was reduced to 5 min in total. The second change was reducing the amount of sample to 0.5 g out of 1 g. The third change was using half the amount of solvents in the extraction process. These solutions reduced the extraction time and the amount of toxic waste while achieving good validation results. A flow chart of the modified QuEChERS procedure is presented in Figure 5.

## 4. Conclusions

The proposed analytical procedures using a modified QuEChERS technique as sample preparation and GC-MS/MS or LC-MS/MS analysis allow for determination of:

- Nineteen and 30 pesticide residues in honeybee samples in a single analytical run using LC and GC analysis, respectively.
- Thirty and 34 pesticide residues in honey samples in a single analytical run using LC and GC analysis, respectively.

Recoveries between 70.1% and 119.4% and coefficient of variation in the range from 0.041 to 20% were obtained. The limits of quantification (LOQ) were in the range from 2.8 to 91 ng/g. Accuracy and precision (in intermediate precision conditions) met the European Community recommendations for pesticide residues in SANCO guidance. The validated methods proved to be fast, efficient and reliable—mainly due to the reduction of the sample weight and the amount of solvents and can be used in the monitoring of pesticides in honey and honeybee samples. 

The use of Eco-Scale allows new insights for the analytical scheme of the developed procedures:

- GC-MS/MS in the Eco-Scale (90 and 82) for honey and honeybee samples, respectively.
- LC-MS/MS in the Eco-Scale (70 and 62) for honey and honeybee samples, respectively.

In conclusion, the proposed QuEChERS extraction meets the requirements of green chemistry. However, despite the fact that the metrological parameters for both of the proposed methods are comparable, it is up to the analyst to choose the appropriate method of determination.

The studies carried out and the data obtained confirm that before final determination, even when sensitive detection is used, the sample preparation and purification step is necessary. 

The developed analytical methods can be helpful in determining the values of the maximum residue levels of pesticides for honeybee samples (that are currently unknown) and in analytical laboratories in which samples are analyzed in case of suspicion of acute honeybee poisoning with plant protection products.

## Figures and Tables

**Table 1:** Expanded uncertainty for selected pesticides.

		LC-MS/MS				GC-MS/MS					
		Honeybee		Honey		Honeybee			Honey		
		MQL	3 × MQL	MQL	3 × MQL	MQL	20	50	MQL	20	50
Alachlor	ng/g	91	273	90	270	10			8.2		
	u_cal_	16%	4.6%	5.5%	1.6%	20%	9.8%	4.0%	5.2%	2.5%	0.93%
	u_recovery_	3.6%	0.41%	2.0%	1.1%	1.4%	2.1%	4.2%	4.7%	0.39%	3.2%
	u_precision_	4.7%	2.4%	1.1%	2.4%	29%	27%	1.9%	20%	0.29%	0.16%
	u_total_	17%	5.2%	6.0%	3.1%	35%	29%	6%	21%	2.5%	3.3%
	U_total_ (k = 2)	34%	10%	12%	6.2%	71%	58%	12%	42%	5.1%	6.7%
Bifenthrin	ng/g	4.9	14.7	4.0	12	8.2	20	50	8.5	20	50
	u_cal_	2.7%	1.3%	4.1%	1.2%	24%	9.2%	3.5%	26%	10%	3.9%
	u_recovery_	9.5%	0.13%	0.89%	2.4%	5.4%	8.9%	8.5%	3.9%	0.80%	2.8%
	u_precision_	0.11%	0.11%	1.1%	0.33%	3.9%	0.26%	0.15%	0.15%	2.1%	6.1%
	u_total_	10%	1.3%	4.3%	2.7%	25%	13%	9.2%	26%	10%	7.7%
	U_total_ (k = 2)	20%	2.6%	8.7%	5.4%	50%	26%	18%	53%	20%	15%
Diazinon	ng/g	4.3	12.9	4.1	12.3	10	20	50	8.3	20	50
	u_cal_	14%	4.1%	6.6%	1.9%	17%	8.1%	3.2%	37%	14%	5.3%
	u_recovery_	6.5%	0.92%	1.7%	0.48%	9.4%	11%	11%	5.6%	5.4%	3.7%
	u_precision_	3.8%	6.4%	4.2%	1.4%	28%	18%	1.9%	32%	15%	2.4%
	u_total_	16%	7.7%	8.0%	2.4%	34.1%	23%	12%	49%	21%	6.9%
	U_total_ (k = 2)	32%	15%	16%	4.8%	68%	46%	23%	98%	42%	14%
Dimoxystrobin	ng/g	4.3	12.9	4.0	12	12	20	50	12	20	50
	u_cal_	6.0%	1.9%	12%	3.5%	14%	7.9%	3.0%	9.1%	5.2%	2.0%
	u_recovery_	3.3%	0.018%	0.33%	1.4%	4.9%	7.6%	6.7%	1.3%	2.6%	0.82%
	u_precision_	3.8%	3.3%	1.1%	0.044%	30%	8.9%	1.7%	1.4%	6.4%	0.15%
	u_total_	7.8%	3.8%	12%	3.8%	33%	14%	7.5%	9.3%	8.6%	2.2%
	U_total_ (k = 2)	16%	7.6%	24%	7.5%	67%	28%	15%	19%	17%	4.3%

**Table 2:** The penalty points for pesticide residues determination in honey and honeybee samples using GC-MS/MS.

Honey Samples		Honeybee Samples	
**REAGENTS**			
	**Penalty Points**		**Penalty Points**
Acetonitrile: 10 mL Acetonitrile: 5 mL	8 ^1^ 4 ^2^	Acetonitrile: 10 mL Acetonitrile: 5 mL	8 ^1^ 4 ^2^
		n-Hexane	8 ^1,2^
	Σ8 ^1^ Σ4 ^2^		Σ16 ^1^ Σ12 ^2^
**INSTRUMENTS ^1,2^**			
	**Penalty Points**		**Penalty Points**
GC-MS/MS	2	GC-MS/MS	2
Waste	1 + 3 = 4	Waste	1 + 3 = 4
	Σ6		Σ6
TOTAL PENALTY POINTS	14 ^1^ 10 ^2^	TOTAL PENALTY POINTS	22 ^1^ 18 ^2^
GREEN ANALYTICAL ECO-SCALE TOTAL SCORE	86 ^1^ 90 ^2^	GREEN ANALYTICAL ECO-SCALE TOTAL SCORE	78 ^1^ 82 ^2^

^1^ before QuEChERS modification; ^2^ after QuEChERS modification.

**Table 3:** The penalty points for pesticide residues determination in honey and honeybee samples using LC-MS/MS.

Honeybee Samples			Honey Samples	
**REAGENTS**				
	**Penalty Points**			**Penalty Points**
Acetonitrile: 10 mL Acetonitrile: 5 mL	8 ^1^ 4 ^2^	Acetonitrile: 10 mL Acetonitrile: 5 mL		8 ^1^ 4 ^2^
n-Hexane	8 ^1,2^			
Mobile phase^1,2^:		Mobile phase ^1,2^:		
Methanol: 10 mL	6	Methanol: 10 mL		6
Acetic Acid: 1 mL	4	Acetic Acid: 1 mL		4
Ammonia: 1 mL	6	Ammonia: 1 mL		6
	Σ32 ^1^ Σ28 ^2^			Σ24 ^1^ Σ20 ^2^
**INSTRUMENTS ^1,2^**				
	**Penalty Points**			**Penalty Points**
LC-MS/MS	2	LC-MS/MS		2
Waste	5 + 3 = 8	Waste		5 + 3 = 8
	Σ10			Σ10
TOTAL PENALTY POINTS	42 ^1^ 38 ^2^	TOTAL PENALTY POINTS		34 ^1^ 30 ^2^
GREEN ANALYTICAL ECO-SCALE TOTAL SCORE	58 ^1^ 62 ^2^	GREEN ANALYTICAL ECO-SCALE TOTAL SCORE		66 ^1^ 70 ^2^

^1^ before QuEChERS modification; ^2^ after QuEChERS modification.

## Supplementary Materials

The following are available online. Table S1: Chemicals and analytical procedures used for determination of pesticide residues, Table S2: Linearity and matrix effects, Table S3: The analytical parameters of the QuEChERS methods in spiked honey and honeybee samples using LC-MS/MS and GC-MS/MS analysis, Table S4: Pesticide residues determined in honey and honeybee samples collected from three counties of Pomerania (concentration with expanded uncertainty). Sample numbers: 1–5 from Gdańsk County, 6–10 from Kartuzy County and 11–15 from Tczew County, Table S5: Comparison of analytical performance of different procedures based on the application of QuEChERS approach with clean-up dSPE step for determination of pesticide residues in honey (A) and honeybee (B) samples.
