# Supplementary material for: Comparison of Two Methods for the Determination of Selected Pesticides in Honey and Honeybee Samples
Source: Molecules. 2018 Oct 9;23(10):2582. doi: 10.3390/molecules23102582 (PMC6222677; doi:10.3390/molecules23102582)
Supplement: Supplementary file 1 [file molecules-23-02582-s001.zip › molecules-366038-SI.pdf]

**SUPPLEMENTARY DATA**

# Comparison of two methods for the determination of selected pesticides in honey and honeybee samples.

**Żaneta Bargańska[1,*], Piotr Konieczka[1] and Jacek Namieśnik [1]**

[1] Department of Analytical Chemistry, Faculty of Chemistry, Gdansk University of Technology, Narutowicza 11/12 str., 80-233 Gdansk, Poland; zanjarz@gmail.com (Ż.B.); piotr.konieczka@pg.gda.pl (P.K.); jacek.namiesnik@pg.edu.pl (J.N.)

* Correspondence: zanjarz@gmail.com; Tel.: +48-58-347-2110; Fax: +48-58-347-2694.

Table 1S. Chemicals and analytical procedures used for determination of pesticide residues.

| **Chemicals and reagents** | |
|---|---|
| Producer/Suplier | Composition |
| Dr Ehrenstorfer GmBH (Germany) - Certified Reference Materials (CRMs) | spinosad, haloxyfrop-R-methyl and dimoxystrobin (in acetonitrile, 10 mg/mL) |
| LGC Standards (Łomianki, Poland) - CRMs | azinphos methyl, azinphos ethyl, bifenthrin, carfentrazone-ethyl, coumaphos, cypermethrin, diazinon, dimethoate, fentrothion, heptenophos, malathion, oxydemeton-methyl, profenofos, pyrazophos, tau-flauvalinate, temephos and vinclozolin (in acetonitrile, 100 mg/mL) |
| Ultra Scientific (North Kingston, RI, USA) - CRMs | alachlor, carbofuran, carbosulfan, chloridazon, indoxacarb (in methanol, 100 mg/mL),<br>imidacloprid, fenoxycarb, fenpyroximate, fenthion, metconazole, methidathion, methiocarb, methomyl, omethoate, oxamyl, pirimicarb, prosulfocarb, quinalphos, triazophos and thiamethoxam (in acetonitrile, 100 mg/mL) |
| Sigma-Aldrich (Schnelldorf, Germany) - CRMs | triphenyl phosphate (TPP) (in methyl tertbutyl ether, 500 mg/mL)<br>aclonifen, ancymidol, chlorpyrifos-methyl, dazomet, dieldrin, endosulfan (alfa isomer), o,p`-DDD, parathion, parathion-methyl, pentachlorophenol, phenthoate, pirimiphos-methyl, prothioconazole, pyriproxyfen and triticonazole (in acetonitrile, 100 mg/mL) |
| Fluka (Sigma-Aldrich, Germany) | acetonitrile, methanol (LC-MS Chromosolv®, ≥99.9%) and n-hexane (Envisolv®, 95%) |
| POCh (Poland) | acetic acid and aqueous ammonia |
| Agilent Technologies (USA) | The QuEChERS kits with salt packets containing 4 g anhydrous magnesium sulfate,1 g sodium chloride,1 g of sodium citrate and 0.5 g sodium hydrogencitrate sesquihydrate, and two-milliliter centrifuge tubes with 150 mg anhydrous magnesium sulfate and 25 mg primary-secondary amine (PSA) for dispersive solid phase extraction (dSPE) |

| **Analitycal procedure** | | | |
|---|---|---|---|
| **LC-MS/MS** | | **GC-MS/MS** | |
| Liquid chromatograph | G4220A | Gas chromatograph | 7890A GC |
| Detector | 6460A<br>(Agilent Technologies, USA) | Detector | 7000 MS/MS<br>(Agilent Technologies, USA) |
| LC column | Poroshell 120 EC-C18<br>(2.7μm; 3×100 mm) | Collision gas | Nitrogen (1.5 ml/min) |
| | | Damping gas | Helium (2.25 ml/min) |
| Column temperaturę | 25°C | Carrier gas | Helium (9.3848 psi) |
| Flow rate | 0.4 ml/min | Injection mode | PTV |
| Volume of injection | 2 μl | Volume of injection | 10 μl |
| Gradient of elution | | GC column | HP-5MS UI<br>(0.25 μm, 0.25 mm × 30 m) |
| Time [min] | $MeOH:H_2O$ [%] | | |
| 0 | 20/80 | Precolumn | quartz capillary tube (1 m |
| 10 | 50/50 | Temperature program | |
| 13.5 | 70/30 | 60°C/min (1 min); 40°C/min do 120°C (0 min); 5°C/min do 310°C (0 min) | |
| 20 | 71/29 | | |
| 29 | 100/0 | | |
| Ionization type | ESI (+) | Ionization type | EI |
| Time of analysis | 35 min | Time of analysis | 40.5 min |
| Calibration | Internal standard | Calibration | Internal standard |

Table 2S. Linearity and matrix effects.

| Compounds | Solvent LC/GC | | LC-MS/MS | | | | | | | | GC-MS/MS | | | | | | | |
|---|---|---|---|---|---|---|---|---|---|---|---|---|---|---|---|---|---|---|
| | | | Honey matrix | | | | Honeybee matrix | | | | Honey matrix | | | | Honeybee matrix | | | |
| | Slope | $R^2$ | Slope | $R^2$ | Slope matrix/ slope solvent | ME [%] = (1−(slope matrix/slope solvent))×100 | Slope | $R^2$ | Slope matrix/ slope solvent | ME [%] = (1−(slope matrix/slope solvent))×100 | Slope | $R^2$ | Slope matrix/ slope solvent | ME [%] = (1−(slope matrix/slope solvent))×100 | Slope | $R^2$ | Slope matrix/ slope solvent | ME [%] = (1−(slope matrix/slope solvent))×100 |
| Aclonifen | 377 | 0.991 | - | | | | | | | | 299 | 0.994 | 0.79 | -21 | 383 | 0.995 | 1.0 | 1.6 |
| Alachlor | 1123/826 | 0.997/0.996 | 1074 | 0.999 | 0.96 | -4.4 | 1154 | 0.991 | 1.0 | 2.8 | 759 | 0.999 | 0.92 | -8.1 | - | | | |
| Ancymidol | 1660 | 0.998 | - | | | | | | | | 1392 | 0.999 | 0.84 | -16 | 1760 | 0.997 | 1.1 | 6.0 |
| Azinphos ethyl | 11355/1413 | 0.998/0.999 | 10868 | 0.998 | 0.96 | -4.3 | - | | | | 1356 | 0.998 | 0.97 | -3.2 | 1723 | 0.99 | 1.2 | 22 |
| Azinphos methyl | 10200 | 0.997 | 9864 | 0.997 | 0.97 | -3.3 | - | | | | - | | | | | | | |
| Bifenthrin | 1676/7538 | 0.990/0.995 | 1586 | 0.999 | 0.95 | -5.4 | 2022 | 0.999 | 1.2 | 21 | 7218 | 0.997 | 0.96 | -4.3 | 8006 | 0.998 | 1.1 | 6.2 |
| Chlorpyrifos- | 7162 | 0.996 | | | | | | | | | 6358 | 0.997 | 0.89 | -11 | 8210 | 0.998 | 1.2 | 15 |
| Chloridazon | 2671 | 0.995 | 2590 | 0.996 | 0.97 | -3.0 | - | | | | | | | | | | | |
| Cypermethrin | 1411 | 0.999 | - | | | | | | | | 1352 | 0.999 | 0.96 | -4.2 | 1341 | 0.998 | 0.95 | -5.0 |
| Dazomet | 467 | 0.999 | | | | | | | | | 360 | 0.991 | 0.77 | -23 | 364 | 0.992 | 0.78 | -22 |
| Diazinon | 1208/847 | 0.999/0.996 | 1111 | 0.998 | 0.92 | -8.0 | 1458 | 0.992 | 1.2 | 21 | 1021 | 0.992 | 1.2 | 21 | - | | | |
| Dieldrin | 142 | 0.996 | | | | | | | | | 148 | 0.997 | 1.0 | 4.1 | 151 | 0.996 | 1.1 | 6.3 |
| Dimethoate | 7166/1191 | 0.996/0.996 | 6580 | 0.996 | 0.92 | -8.2 | 7452 | 0.998 | 1.0 | 4.0 | 1124 | 0.994 | 0.94 | -5.6 | 1554 | 0.996 | 1.3 | 31 |
| Dimoxystrobin | 19643/5616 | 0.996/0.998 | 19046 | 0.996 | 0.97 | -3.0 | 16941 | 0.998 | 0.86 | -14 | 4527 | 0.996 | 0.81 | -19 | 6129 | 0.997 | 1.1 | 9.1 |
| Endosulfan (alfa | 59 | 0.997 | - | | | | | | | | 50 | 0.998 | 0.83 | -17 | 69 | 0.998 | 1.2 | 16 |
| Fenitrothion | 6520 | 0.994 | - | | | | | | | | 6267 | 0.997 | 0.96 | -3.9 | 6467 | 0.997 | 0.99 | -0.81 |
| Fenthion | 6466 | 0.997 | - | | | | | | | | - | | | | 7105 | 0.996 | 1.1 | 9.9 |
| Fenoxycarb | 13373 | 0.996 | - | | | | 18216 | 0.997 | 1.4 | 36 | - | | | | | | | |
| Fenpyroximate | 15351 | 0.997 | 15582 | 0.999 | 1.0 | 1.5 | 18596 | 0.996 | 1.2 | 21 | - | | | | | | | |

| | | | | | | | | | | | | | | | | | | |
|---|---|---|---|---|---|---|---|---|---|---|---|---|---|---|---|---|---|---|
| Haloxyfop-R-methyl | 8502/407 | 0.998/0.998 | 8300 | 0.996 | 0.98 | -2.4 | - | | | | 398 | 0.997 | 0.98 | -2.1 | 465 | 0.998 | 1.1 | 14 |
| Heptenophos | 1890/353 | 0.997/0.996 | 1704 | 0.998 | 0.90 | -9.9 | 1350 | 0.997 | 0.71 | -29 | 286 | 0.993 | 0.81 | -19 | 245 | 0.993 | 0.69 | -31 |
| Imidacloprid | 6758 | 0.998 | 6434 | 0.999 | 0.95 | -4.8 | - | | | | - | | | | | | | |
| Indoxacarb | 2321 | 0.998 | 2319 | 0.995 | 0.99 | -0.10 | 2842 | 0.992 | 1.2 | 22 | - | | | | | | | |
| Carbofuran | 13803 | 0.994 | - | | | | 16387 | 0.995 | 1.2 | 19 | - | | | | | | | |
| Carbosulfan | 508 | 0.997 | - | | | | | | | | 538 | 0.997 | 1.1 | 6.0 | - | | | |
| Carfetrazone-ethyl | 3814 | 0.997 | 3585 | 0.999 | 0.94 | -6.0 | 4969 | 0.998 | 1.3 | 30 | - | | | | | | | |
| Coumaphos | 11509 | 0.998 | 10958 | 0.999 | 0.95 | -4.8 | 12963 | 0.999 | 1.1 | 13 | - | | | | | | | |
| Quinalphos | 3440/2743 | 0.997/0.997 | 3265 | 0.996 | 0.95 | -5.1 | - | | | | 2432 | 0.998 | 0.89 | -11 | 3121 | 0.997 | 1.1 | 14 |
| Malathion | 2230 | 0.997 | - | | | | | | | | 2305 | 0.997 | 1.0 | 3.4 | 2196 | 0.997 | 0.98 | -1.5 |
| Metconazole | 41 | 0.997 | - | | | | | | | | 41 | 0.998 | 1.0 | -0.24 | 50 | 0.998 | 1.2 | 21 |
| Methiocarb | 2050 | 0.996 | 1931 | 0.997 | 0.94 | -5.8 | - | | | | - | | | | | | | |
| Methomyl | 3410 | 0.998 | 3268 | 0.998 | 0.96 | -4.2 | - | | | | - | | | | | | | |
| Methidathion | 1224/1596 | 0.998/0.993 | 1155 | 0.999 | 0.94 | -5.6 | 1521 | 0.997 | 1.2 | 24 | 1206 | 0.996 | 0.76 | -24 | 2003 | 0.995 | 1.3 | 26 |
| o,p'-DDD | 2694 | 0.997 | - | | | | | | | | 2754 | 0.997 | 1.0 | 2.2 | 2575 | 0.997 | 0.96 | -4.5 |
| Oxamyl | 8044 | 0.990 | 7634 | 0.996 | 0.95 | -5.1 | - | | | | - | | | | | | | |
| Oxydemeton-methyl | 10019 | 0.995 | 9663 | 0.999 | 0.96 | -3.6 | 13651 | 0.997 | 1.4 | 36 | - | | | | | | | |
| Omethoate | 9036 | 0.990 | 8258 | 0.996 | 0.91 | -8.6 | 8599 | 0.997 | 0.95 | -4.8 | - | | | | | | | |
| Phenthoate | 600 | 0.994 | - | | | | | | | | 590 | 0.995 | 0.98 | -1.7 | - | | | |
| Parathion | 525 | 0.998 | - | | | | | | | | 615 | 0.998 | 1.2 | 17 | 626 | 0.998 | 1.2 | 19 |
| Parathion-methyl | 6588 | 0.997 | - | | | | | | | | - | | | | 7108 | 0.997 | 1.1 | 7.9 |
| Pentachlorophenol | 64 | 0.997 | - | | | | | | | | 67 | 0.998 | 1.1 | 5.0 | 68 | 0.998 | 1.1 | 6.9 |
| Pirimiphos-methyl | 109 | 0.996 | - | | | | | | | | 95 | 0.997 | 0.87 | -13 | 119 | 0.996 | 1.1 | 9.8 |
| Pirimicarb | 11831 | 0.996 | 11776 | 0.990 | 0.99 | -0.46 | - | | | | - | | | | | | | |
| Profenofos | 1955 | 0.998 | 1862 | 0.999 | 0.95 | -4.8 | 2704 | 0.997 | 1.4 | 38 | - | | | | | | | |
| Prosulfocarb | 1980 | 0.998 | - | | | | | | | | 1827 | 0.999 | 0.92 | -7.7 | 1999 | 0.998 | 1.0 | 0.96 |

| | | | | | | | | | | | | | | | | | | |
|---|---|---|---|---|---|---|---|---|---|---|---|---|---|---|---|---|---|---|
| Prothioconazole | 986 | 0.996 | - | | | | | | | | 970 | 0.997 | 0.98 | -1.7 | - | | | |
| Pyrazophos | 14111/848 | 0.997/0.998 | 13483 | 0.998 | 0.96 | -4.5 | 17568 | 0.998 | 1.3 | 25 | 785 | 0.998 | 0.93 | -7.5 | 973 | 0.998 | 1.2 | 15 |
| Pyriproxyfen | 2527 | 0.995 | - | | | | | | | | 2431 | 0.996 | 0.96 | -3.8 | 2642 | 0.997 | 1.1 | 4.6 |
| Spinosad A | 23948 | 0.999 | 22640 | 0.999 | 0.95 | -5.5 | - | | | | - | | | | | | | |
| Spinosad D | 5398 | 0.999 | 5100 | 0.999 | 0.95 | -5.5 | - | | | | - | | | | | | | |
| Tau-fluvalinate | 1411 | 0.999 | - | | | | | | | | 1454 | 0.999 | 1.03 | 3.0 | 1423 | 0.999 | 1.0 | 0.85 |
| Temephos | 7195 | 0.997 | 7000 | 0.996 | 0.97 | -2.7 | 8984 | 0.998 | 1.3 | 25 | - | | | | | | | |
| Thiamethoxam | 8514 | 0.999 | 8172 | 0.998 | 0.96 | -4.0 | 9753 | 0.995 | 1.2 | 15 | - | | | | | | | |
| Triazophos | 31518/800 | 0.995/0.997 | 30658 | 0.997 | 0.97 | -2.7 | - | | | | 787 | 0.995 | 0.98 | -1.7 | 823 | 0.996 | 1.0 | 2.8 |
| Triticonazole | 218 | 0.996 | - | | | | | | | | 213 | 0.998 | 0.98 | -2.1 | - | | | |
| Vinclozolin | 435 | 0.997 | - | | | | | | | | 472 | 0.997 | 1.1 | 8.6 | 493 | 0.996 | 1.1 | 14 |

Table 3S. The analytical parameters of the QuEChERS methods in spiked honey and honeybee samples using LC-MS/MS and GC-MS/MS analysis.

| | | LC-MS/MS | | | | | | GC-MS/MS | | | | | | | |
|---|---|---|---|---|---|---|---|---|---|---|---|---|---|---|---|
| | | Honeybee | | | Honey | | | Honeybee | | | | Honey | | | |
| MRLs for honey [ng/g] [1] | Compound | LOQ [ng/g] | Recovery (CV) [%] (n=5) | | MQL [ng/g] | Recovery (CV) [%] (n=5) | | LOQ [ng/g] | Recovery (CV) [%] (n=5) | | | MQL [ng/g] | Recovery (CV) [%] (n=5) | | |
| | | | 3LOQ | LOQ | | 3LOQ | LOQ | | LOQ | 20 [ng/g] | 50 [ng/g] | | LOQ | 20 [ng/g] | 50 [ng/g] |
| 50 | Aclonifen | - | | | | | | 9.7 | 101 (8) | 101 (13) | 100 (6) | 10 | 94.7 (2.6) | 96.5 (9.7) | 100 (2) |
| 10 | Alachlor | 91 | 83.34 (0.77) | 81.5 (6.5) | 90 | 84.0 (2.1) | 83.1 (3.7) | - | | | | 8.2 | 95 (10) | 100.00 (0.88) | 99.0 (7.1) |
| - | Ancymidol | - | | | | | | 9.5 | 100 (13) | 99.9 (9.4) | 100 (13) | 9.9 | 92.0 (5.1) | 97 (19) | 100.0 (5.6) |
| - | Azinphos ethyl | - | | | 4.6 | 95.13 (0.66) | 97.22 (0.79) | 12 | 100 (13) | 101 (18) | 100.0 (8.9) | 12 | 94.5 (3.0) | 99.8 (3.8) | 100.0 (2.1) |
| - | Azinphos methyl | - | | | 4.0 | 95.0 (2.4) | 95.8 (1.6) | - | | | | | | | |
| 50 | Bifenthrin | 4.9 | 76.08 (0.22) | 85 (18) | 4.0 | 98.3 (5.3) | 100.0 (2.0) | 8.2 | 100 (12) | 100 (20) | 100 (19) | 8.5 | 95.2 (8.4) | 100.1 (1.8) | 95.6 (5.9) |
| - | Chlorpyrifos-methyl | - | | | | | | 12 | 100.0 (2.8) | 92 (20) | 109 (15) | 13 | 92 (20) | 90 (20) | 90 (19) |
| 10 | Chloridazon | - | | | 5.4 | 80.8 (2.0) | 81.3 (2.0) | - | | | | | | | |
| 50 | Cypermethrin | - | | | | | | 8.2 | 100.8 (4.6) | 99.2 (8.8) | 99.7 (4.6) | 8.8 | 85.5 (6.9) | 100.0 (9.1) | 94.4 (6.5) |
| 50 | Dazomet | - | | | | | | 12 | 100.21 (0.92) | 93 (18) | 95 (12) | 12 | 96.2 (7.4) | 100.0 (5.4) | 98.9 (5.2) |
| 10 | Diazinon | 4.3 | 82.7 (1.7) | 103 (15) | 4.1 | 96.88 (1.03) | 97.0 (3.7) | - | | | | 8.3 | 96 (12) | 100 (12) | 98.2 (8.2) |
| 10 | Dieldrin | - | | | | | | 11 | 102.12 (0.47) | 100.4 (1.3) | 114.0 (3.1) | 12 | 96 (11) | 100 (12) | 102.2 (7.7) |
| - | Dimethoate | 4.1 | 85.63 (0.28) | 85.3 (7.2) | 3.8 | 91.0 (1.6) | 91.70 (0.84) | 8.5 | 100.0 (7.9) | 97 (18) | 97 (11) | 8.5 | 95.4 (8.9) | 100.0 (1.1) | 98.7 (6.3) |
| 50 | Dimoxystrobin | 4.3 | 104.451 (0.041) | 76.8 (5.7) | 4.0 | 96.2 (3.0) | 98.81 (0.73) | 12 | 100 (11) | 100 (17) | 100 (15) | 12 | 94.9 (2.7) | 100.0 (5.8) | 103.5 (1.9) |
| 10 | Endosulfan (alfa isomer) | - | | | | | | 7.9 | 100 (12) | 100.0 (6.8) | 112.1 (4.2) | 8.3 | 95.3 (9.3) | 100.0 (8.0) | 99.8 (6.6) |
| 10 | Fenitrothion | - | | | | | | 8.2 | 100.0 (2.7) | 91 (19) | 97 (18) | 9.0 | 94 (19) | 90 (18) | 89 (18) |
| 10 | Fenthion | - | | | | | | 10 | 100.0 (2.8) | 93 (18) | 96 (14) | - | | | |

| | | | | | | | | | | | | | | | |
|---|---|---|---|---|---|---|---|---|---|---|---|---|---|---|---|
| 50 | Fenoxycarb | 4.5 | 91.9 (1.3) | 70.8 (6.3) | - | | | - | | | | | | | |
| 50 | Fenpyroxymate | 4.1 | 97.0 (1.5) | 83.00 (0.76) | 3.6 | 99 (15) | 108.1 (1.4) | - | | | | | | | |
| - | Phenthoate | - | | | | | | 23 | 94.9 (8.1) | 100.0 (5.6) | 100.0 (5.7) | - | | | |
| 50 | Haloxyfop-R-methyl | - | | | 3.7 | 92.7 (4.0) | 90.53 (0.54) | 13 | 100.3 (1.1) | 100.56 (0.85) | 99.94 (0.74) | 13 | 96 (11) | 100.1 (1.5) | 103.4 (7.7) |
| - | Heptenophos | 3.2 | 94.631 (0.074) | 93.0 (8.0) | 3.1 | 96.5 (1.2) | 98.2 (2.3) | 8.1 | 100.0 (2.0) | 85 (16) | 97 (14) | 7.7 | 94 (20) | 100.00 (0.14) | 101 (14) |
| 50 | Imidacloprid | - | | | 4.1 | 92.2 (6.2) | 95.0 (1.5) | - | | | | | | | |
| 50 | Indoxacarb | 3.9 | 119.4 (1.6) | 70.8 (5.1) | 4.0 | 90.2 (6.5) | 95.25 (0.94) | - | | | | | | | |
| 50 | Carbofuran | 2.8 | 106.1 (1.2) | 109 (11) | - | | | - | | | | | | | |
| - | Carbosulfan | - | | | | | | - | | | | 5.7 | 97.2 (3.5) | 100.0 (1.3) | 102.0 (2.5) |
| - | Carfentrazone-ethyl | 4.3 | 99.48 (0.45) | 78.9 (8.9) | 4.0 | 94 (6.0) | 98.1 (3.4) | - | | | | | | | |
| 100 | Coumaphos | 6.4 | 76.18 (0.95) | 80.4 (7.7) | 5.1 | 96.2 (4.5) | 100 (2.0) | - | | | | | | | |
| 50 | Quinalphos | - | | | 4.2 | 91.3 (3.1) | 89.59 (0.32) | 15 | 100.7 (1.3) | 100 (12) | 99.5 (8.8) | 15 | 96 (11) | 100.0 (3.6) | 100.0 (7.7) |
| 50 | Malathion | - | | | | | | 16 | 99.7 (5.3) | 100.3 (3.8) | 90 (19) | 15 | 95.8 (9.7) | 100.0 (2.2) | 99.0 (6.9) |
| 50 | Methiocarb | - | | | 3.8 | 91.78 (0.67) | 92.13 (1.17) | - | | | | | | | |
| 50 | Metconazole | - | | | | | | 12 | 94 (19) | 100 (15) | 93 (13) | 12 | 94.8 (3.2) | 100.0 (4.2) | 93.1 (2.3) |
| 10 | Methomyl | - | | | 4.4 | 80.8 (1.5) | 82.36 (1.05) | - | | | | | | | |
| 20 | Methidathion | 5.0 | 106.8 (1.8) | 74.6 (6.7) | 5.0 | 96.5 (2.9) | 97.6 (1.8) | 3.7 | 100 (13) | 100 (15) | 101 (13) | 3.8 | 95.4 (10.0) | 100.00 (0.89) | 102.4 (7.1) |
| 50 | o.p'-DDD | - | | | | | | 11 | 100.0 (2.0) | 100.1 (2.9) | 109.1 (1.4) | 11 | 95.1 (9.1) | 100.0 (4.8) | 98.2 (6.4) |
| 50 | Oxamyl | - | | | 2.8 | 83.2 (2.4) | 84.01 (0.59) | - | | | | | | | |
| 10 | Oxydemeton-methyl | 3.4 | 103.9 (2.3) | 87.3 (9.3) | 3.1 | 86.0 (1.3) | 86.06 (0.39) | - | | | | | | | |
| - | Omethoate | 3.3 | 110.58 (0.14) | 71.6 (2.1) | 2.9 | 81.10 (0.42) | 80.69 (0.44) | - | | | | | | | |
| - | Parathion | - | | | | | | 11 | 100 (19) | 100.1 (5.4) | 113 (14) | 8.0 | 96.1 (2.4) | 100.0 (5.3) | 101.2 (1.7) |

| | | | | | | | | | | | | | | | |
|---|---|---|---|---|---|---|---|---|---|---|---|---|---|---|---|
| 10 | Parathion-methyl | - | | | | | | 14 | 100.0 (2.8) | 95 (20) | 98 (18) | - | | | |
| - | Pentachlorophenol | - | | | | | | 29 | 99 (20) | 100.0 (6.6) | 111.1 (5.5) | 27 | 92.3 (9.5) | 100 (16) | 97 (14) |
| 50 | Pirimiphos-methyl | - | | | | | | 14 | 100.0 (1.2) | 100.00 (0.83) | 100.1 (1.8) | 14 | 97 (16) | 100.0 (2.0) | 99 (11) |
| 50 | Pirimicarb | - | | | 3.6 | 92.6 (1.7) | 94.85 (0.51) | - | | | | | | | |
| 50 | Profenofos | 3.2 | 109.1 (1.7) | 80.1 (5.7) | 3.4 | 92.6 (4.3) | 108.6 (1.2) | - | | | | | | | |
| 50 | Prosulfocarb | - | | | | | | 11 | 102.2 (1.4) | 103.5 (7.6) | 106.2 (2.6) | 11 | 97 (15) | 100.00 (0.21) | 101 (11) |
| 50 | Prothioconazole | - | | | | | | - | | | | 11 | 95.2 (7.2) | 100.0 (3.6) | 97.1 (5.1) |
| 50 | Pyrazophos | 4.5 | 98.69 (0.32) | 70.2 (1.9) | 4.3 | 92.1 (4.4) | 90.11 (0.57) | 3.6 | 100 (15) | 100 (19) | 100.2 (15.1)2 | 3.7 | 94.3 (5.3) | 100.0 (7.3) | 98.2 (3.7) |
| 50 | Pyriproxyfen | - | | | | | | 7.8 | 100 (19) | 95 (19) | 99 (19) | 7.9 | 95.0 (4.7) | 100.0 (7.0) | 100.0 (3.4) |
| 50 | Spinosad A | - | | | 2.9 | 100.3 (6.1) | 106.18 (0.90) | - | | | | | | | |
| | Spinosad D | - | | | 2.8 | 106 (10) | 109.5 (2.9) | - | | | | | | | |
| 50 | tau-flauvalinate | - | | | | | | 15 | 112 (10) | 102 (17) | 116 (14) | 16 | 94 (16) | 99.2 (1.4) | 100.0 (1.0) |
| - | Temephos | 4.8 | 81.47 (1.20) | 72.70 (0.36) | 3.7 | 96.6 (10.0) | 102.97 (0.79) | - | | | | | | | |
| 50 | Thiamethoxam | 4.7 | 74.38 (0.92) | 70.1 (2.0) | 4.0 | 84.5 (2.4) | 85.5 (1.8) | - | | | | | | | |
| 50 | Triazophos | - | | | 3.9 | 90.9 (1.6) | 90.9 (1.4) | 2.2 | 100 (17) | 100.1 (6.6) | 100 (12) | 2.3 | 95.3 (6.9) | 98.1 (4.1) | 100.0 (4.9) |
| 10 | Triticonazole | - | | | | | | - | | | | 13 | 93.3 (2.5) | 103.5 (8.0) | 98.2 (1.8) |
| 50 | Vinclozolin | - | | | | | | 15 | 100.0 (4.3) | 101 (14) | 104 (13) | 15 | 96.0 (7.4) | 100.0 (5.3) | 90.0 (6.8) |

Table 4S. Pesticide residues determined in honey and honeybee samples collected from three counties of Pomerania (concentration with expanded uncertainty). Sample numbers: 1-5 from Gdańsk County, 6-10 from Kartuzy County and 11-15 from Tczew County.

| | | | GC-MS/MS | | | LC-MS/MS | | | GC-MS/MS | | | LC-MS/MS | | |
|---|---|---|---|---|---|---|---|---|---|---|---|---|---|---|
| | | | **Honey samples** | | | | | | **Honeybee samples** | | | | | |
| **Pesticides** | **GC/LC** | **MRLs [1]** | **Number of samples (positive)** | **Min level [ng/g] (RSD)** | **Max level [ng/g] (RSD)** | **Number of samples (positive)** | **Min level [ng/g] (RSD)** | **Max level [ng/g] (RSD)** | **Number of samples (positive)** | **Min level [ng/g] (RSD)** | **Max level [ng/g] (RSD)** | **Number of samples (positive)** | **Min level [ng/g] (RSD)** | **Max level [ng/g] (RSD)** |
| Aclonifen | GC | 50 | - | nd | nd | - | nd | nd | 4-10 | <LOQ | <LOQ | - | nd | nd |
| Alachlor | GC/LC | 10 | - | nd | nd | 4,10,11,14 | <LOQ | <LOQ | - | nd | nd | 1,11 | <LOQ | 97.2 (9.3) |
| Ancymidol | GC | - | 1,4,15 | <LOQ | 13.64 (0.13) | - | nd | nd | 1-10 | <LOQ | 56.32 (1.38) | - | nd | nd |
| Azinphos ethyl | GC/LC | - | 1-15 | nd | <LOQ | 4,10,11,14 | <LOQ | 26.21 (1.22) | 1-5,8,15 | 14.59 (1.34) | 33.5 (1.8) | - | nd | nd |
| Azinphos methyl | LC | - | - | nd | nd | 2,4,10 | <LOQ | 23.1 (0.9) | - | nd | nd | - | nd | nd |
| Bifenthrin | GC/LC | 50 | - | nd | nd | 4,10,12 | <LOQ | 15.12 (1.1) | 1-10 | nd | <LOQ | 1,5,6,7,10-15 | <LOQ | <LOQ |
| Chlorpyrifos-methyl | GC | - | 1,2,3,5-15 | <LOQ | 20.10 (1.31) | - | nd | nd | 5-10 | <LOQ | 44.12 (2.26) | - | nd | nd |
| Chloridazon | LC | 10 | - | nd | nd | 4,10,12,14 | <LOQ | 7.7 (0.9) | - | nd | nd | - | nd | nd |
| Cypermethrin | GC | 50 | 1-15 | <LOQ | 54.32 (1.10) | - | nd | nd | 1-15 | <LOQ | 24.3 (1.2) | - | nd | nd |
| Dazomet | GC | 50 | 1-15 | nd | <LOQ | - | nd | nd | 1-15 | <LOQ | 13.81 (0.93) | - | nd | nd |
| Diazinon | GC/LC | 10 | - | nd | nd | 4,6,8,10,11,12,14 | <LOQ | <LOQ | - | nd | nd | 1,5,11,12,13,15 | <LOQ | 14.2 (2.2) |
| Dieldrin | GC | 10 | - | nd | nd | - | nd | nd | - | nd | nd | - | nd | nd |
| Dimethoate | GC/LC | - | 1-15 | <LOQ | <LOQ | 4,10,12,14 | <LOQ | <LOQ | 1-15 | <LOQ | <LOQ | 1,8,13,14,15 | <LOQ | 24.15 (2.12) |
| Dimoxystrobin | GC/LC | 50 | - | nd | nd | 3,4,5,6,7,8,10,11,12,14 | <LOQ | <LOQ | 1-10 | <LOQ | 14.7 (1.4) | 11 | <LOQ | <LOQ |
| Endosulfan (alfa isomer) | GC | 10 | - | nd | nd | - | nd | nd | 1-15 | <LOQ | 83.2 (1.9) | - | nd | nd |
| Fenitrothion | GC | 10 | 1-6 | nd | <LOQ | - | nd | nd | 5-10 | nd | <LOQ | - | nd | nd |

| | | | | | | | | | | | | | | |
|---|---|---|---|---|---|---|---|---|---|---|---|---|---|---|
| Fenthion | LC | 10 | - | nd | nd | - | nd | nd | 2-10 | <LOQ | 45.6 (2.5) | - | nd | nd |
| Fenoxycarb | LC | 50 | - | nd | nd | - | nd | nd | - | nd | nd | 11 | nd | 17.2 (1.6) |
| Fenpyroxymate | GC | 50 | - | nd | nd | 4,10,12,14 | <LOQ | <LOQ | - | nd | nd | 1,11 | <LOQ | <LOQ |
| Phenthoate | GC | - | 1 | nd | <LOQ | - | nd | nd | - | nd | nd | - | nd | nd |
| Haloxyfop-R-methyl | GC/LC | 50 | - | nd | nd | 4,10,12,14 | <LOQ | <LOQ | 1-8 | <LOQ | 73.3 (1.4) | - | nd | nd |
| Heptenophos | GC/LC | - | 1,2,7,10,12 | <LOQ | 13.0 (2.1) | 4,10,12,14 | <LOQ | <LOQ | 1-15 | <LOQ | 11.88 (1.23) | 1,2,3,6,8-15 | <LOQ | 19.21 (0.94) |
| Imidacloprid | LC | 50 | - | nd | nd | 4,10,12,14 | <LOQ | <LOQ | - | nd | nd | - | nd | nd |
| Indoxacarb | LC | 50 | - | nd | nd | 4,10,12,14 | <LOQ | <LOQ | - | nd | nd | 11,12 | <LOQ | 13.58 (1.21) |
| Carbofuran | LC | 50 | - | nd | nd | - | nd | nd | - | nd | nd | 1,11 | nd | <LOQ |
| Carbosulfan | GC | - | 1,2,3,5,6, 10-14 | <LOQ | <LOQ | - | nd | nd | - | nd | nd | - | nd | nd |
| Carfentrazone-ethyl | LC | - | - | nd | nd | 4,10,12,14 | <LOQ | 21.13 (2.35) | - | nd | nd | 1,11,14,15 | <LOQ | 24.12 (2.71) |
| Coumaphos | LC | 100 | - | nd | nd | 4,10,11,1,14 | <LOQ | 17.2 (0.9) | - | nd | nd | 11 | nd | <LOQ |
| Quinalphos | GC/LC | 50 | - | nd | nd | 4,5,10,12,14 | <LOQ | 21.2 (1.2) | 5-11 | nd | <LOQ | - | nd | nd |
| Malathion | GC | 50 | 15 | nd | <LOQ | - | nd | nd | 5,15 | nd | <LOQ | - | nd | nd |
| Methiocarb | LC | 50 | - | nd | nd | 4,10,12,14 | <LOQ | 20.0 (1.1) | - | nd | nd | - | nd | nd |
| Metconazole | GC | 50 | 15 | nd | 65.03 (3.02) | - | nd | nd | - | nd | nd | - | nd | nd |
| Methomyl | LC | 10 | - | nd | nd | 4,10,12,14 | <LOQ | 19.21 (0.89) | - | nd | nd | - | nd | nd |
| Methidathion | GC/LC | 20 | 1-15 | <LOQ | <LOQ | 4,10,12,14 | <LOQ | 27.1 (1.3) | 1-15 | nd | <LOQ | 1,5,6,10-15 | <LOQ | 24.3 (1.9) |
| o.p'-DDD | GC | 50 | - | nd | nd | - | nd | nd | - | nd | nd | - | nd | nd |
| Oxamyl | LC | 50 | - | nd | nd | 4,10,12,14 | <LOQ | 16.5 (0.9) | - | nd | nd | - | nd | nd |
| Oxydemeton-methyl | LC | 10 | - | nd | nd | 4,10,12,14 | <LOQ | 16.44 (0.89) | - | nd | nd | 11 | <LOQ | 20.0 (1.2) |
| Omethoate | LC | - | - | nd | nd | 4,10,12,14 | <LOQ | 19.4 (1.3) | - | nd | nd | 11,14,15 | <LOQ | 16.3 (3.1) |
| Parathion | GC | - | - | nd | nd | - | nd | nd | - | nd | nd | - | nd | nd |

| | | | | | | | | | | | | | | |
|---|---|---|---|---|---|---|---|---|---|---|---|---|---|---|
| Parathion-methyl | GC | 10 | - | nd | nd | - | nd | nd | 5-10 | <LOQ | 42.9 (1.3) | - | nd | nd |
| Pentachlorophenol | GC | - | 3,5,6,10,11 | nd | <LOQ | - | nd | nd | 1-4,7,9,10 | nd | <LOQ | - | nd | nd |
| Pirimiphos-methyl | GC | 50 | - | nd | nd | - | nd | nd | - | nd | nd | - | nd | nd |
| Pirimicarb | LC | 50 | - | nd | nd | 4,10,12,14 | <LOQ | 23.14 (1.12) | - | nd | nd | - | nd | nd |
| Profenofos | LC | 50 | - | nd | nd | 1,2,3,4,6,7,10, 11,12,14 | <LOQ | 18.3 (0.8) | - | nd | nd | 2,5,6,10, 11,15 | <LOQ | 10,1 (2.7) |
| Prosulfocarb | GC | 50 | 15 | nd | 14.38 (0.16) | - | nd | nd | 1-15 | <LOQ | 45.6 (2.2) | - | nd | nd |
| Prothioconazole | GC | 50 | - | nd | nd | - | nd | nd | - | nd | nd | - | nd | nd |
| Pyrazophos | GC/LC | 50 | 1,2,3,8,9, 11,15 | <LOQ | 9.76 (0.15) | 4,10,12,14 | <LOQ | 16.3 (1.1) | 1-15 | <LOQ | 46.38 (1.20) | - | nd | nd |
| Pyriproxyfen | GC | 50 | 3,4 | nd | <LOQ | - | nd | nd | 1-10 | <LOQ | <LOQ | - | nd | nd |
| Spinosad | LC | 50 | - | nd | nd | 4,10,12,14 | <LOQ | 21.26 (0.75) | - | nd | nd | - | nd | nd |
| tau-flauvalinate | GC | 50 | - | nd | nd | - | nd | nd | 2-5,8-10,14 | nd | <LOQ | - | nd | nd |
| Temephos | LC | - | - | nd | nd | 4,10,12,14 | <LOQ | 21.58 (0.76) | - | nd | nd | 1,2,13 | <LOQ | <LOQ |
| Thiamethoxam | LC | 50 | - | nd | nd | 4,10,12,14 | <LOQ | 25.2 (1.3) | - | nd | nd | 11,14 | <LOQ | 11.0 (3.0) |
| Triazophos | GC/LC | 50 | 1-15 | nd | <LOQ | 2,4,10,12,14 | <LOQ | 19.25 (0.95) | 1-9 | nd | <LOQ | - | nd | nd |
| Triticonazole | GC | 10 | - | nd | nd | - | nd | nd | - | nd | nd | - | nd | nd |
| Vinclozolin | GC | 50 | - | nd | nd | - | nd | nd | - | nd | nd | - | nd | nd |

nd - not detected

Table 5S. Comparison of analytical performance of different procedures based on the application of QuEChERS approach with clean-up dSPE step for determination of pesticide residues in honey (A) and honeybee (B) samples.

| Type of sample | Compounds | Mass of sample [g] | Time of sample preparation [min] | Selected validation parameters | Final determination technique | Time of analysis [min] | The penalty points granted using EcoScale | References |
|---|---|---|---|---|---|---|---|---|
| B | 150 multiclass pesticides | 2 | ~150 | LOQ: -<br>CV:≤20 [%]<br>Recovery: 47-92 [%]<br>Matrix effects: Average matrix effects exceeding 20% (mainly enhancement) were observed for approximately 33% of the compounds. | GC-MS/MS | ~31 | 86 | [2] |
| A | fipronil, imidacloprid, thiamethoxam, dimethoate, carbendazin, tebuconazole, amitraz, τ-fluvalinate and 5-hydroxy methylfurfural (HMF) | 10 | 13 | LOQ: 5-1000 [ng/g]<br>CV:≤20 [%]<br>Recovery: 86,7-112 [%]<br>Matrix effects: -50 to 200 [%] | LC-APCI-MS/MS | 15 | 70 | [3] |
| B | 54 multiclass pesticides | 1 | 14 | LOD: ≤2 [ng/mL]<br>CV:≤22 [%]<br>Recovery: 58-120 [%]<br>Matrix effects: -92 to 52 [%] | UHPLC-LTQ-Orbitrap-MS | 15 | 64 | [4] |
| A | 52 multiclass pesticides | 5 | ~10 | LOQ: 0.2-10 [ng/g]<br>CV: <20 [%]<br>Recovery: 30-90 [%]<br>Matrix effects: -60 to 50 [%] | LC-MS | 20 | 68 | [5] |
| B | | | | LOQ: 0.03-10 [ng/g]<br>CV: <20 [%]<br>Recovery: 34-96 [%]<br>Matrix effects: -60 to 35 [%] | | | 64 | |
| A | cymiazole, fipronil, coumaphos, fluvalinate, amitraz, and its metabolite 2,4-dimethylaniline (2,4-DMA) | 2 | 44 | LOQ: 1-5 [ng/g]<br>CV: <12 [%]<br>Recovery: 62.06–108.79 [%]<br>Matrix effects: -41.1 to 71 [%] | LC-MS/MS | 10 | 68 | [6] |

| | | | | | | | | |
|---|---|---|---|---|---|---|---|---|
| A | 80 environmental contaminants, pesticides and veterinary drugs, belonging to different chemical classes | 5 | ~5 | LOQ: 3-65.8 [ng/g]<br>CV: ≤20 [%]<br>Recovery:<br>60-120 [%]<br>Matrix effects: - | LC-MS/MS or GC-TOF | 31 | 64 or 90 | [7] |
| B | | 10 | | | | 30 | 60 or 78 | |
| A | 19 multiclass pesticides | 5 | - | LOQ: 0.1-10 [ng/g]<br>CV:≤20 [%]<br>Recovery:<br>70-120 [%]<br>Matrix effects:<br>-50 to 43 [%] | LC-MS/MS | 35 | 73 | [8] |
| B | | 2 | 12.5 | | | | | |
| A | 19 and 30 multiclass pesticides | 0.5 | ~5 | LOQ: 2.8-91 [ng/g]<br>CV:≤20 [%]<br>Recovery:<br>70.1-119.4 [%]<br>Matrix effects:<br>-22.4 to 24.3 [%] | LC-MS/MS | 35 | 70 and 62 for honey and honeybee samples | **This work** |
| B | 30 and 34 multiclass pesticides | | | | GC-MS/MS | 40.5 | 90 and 82 for honey and honeybee samples | |

References:

[1] Regulation EC No 396/2005 of the European Parliament and of the Council of 23 February 2005 on maximum residue levels of pesticides in or on food and feed of plant and animal origin and amending Council Directive 91/414/EEC. Available from:

https://ec.europa.eu/food/plant/pesticides/max_residue_levels/eu_rules_en (accessed 10.07.2018).

[2] Walorczyk, S.; Gnusowski, B. (2009). Development and validation of a multi-residue method for the determination of pesticides in honeybees using acetonitrile-based extraction and gas chromatography-tandem quadrupole mass spectrometry. *Journal of Chromatography A*, 1216, 6522-6531.

[3] Tomasini, D.; Sampaio, M. R. F.; Caldas, S. S.; Buffon, J.G.; Duarte, F.A.; Primel, E.G. (2012). Simultaneous determination of pesticides and 5-hydroxymethylfurfural in honey by the modified QuEChERS method and liquid chromatography coupled to tandem mass spectrometry. *Talanta*, 99, 380-386.

[4] Farré, M.; Picó, Y.; Barceló, D. (2014). Application of ultra-high pressure liquid chromatography linear ion-trap orbitrap to qualitative and quantitative assessment of pesticide residues. *Journal of Chromatography A*, 1328, 66-79.

[5] Calatayud-Vernich, P.; Calatayud, F.; Simó, E.; Picó, Y. (2016). Efficiency of QuEChERS approach for determining 52 pesticide residues in honey and honeybees. *Methods X*, 3, 452-458.

[6] Zheng, W.; Park, J-A.; Abd El-Aty, A.M.; Kim, S-K.; Cho, S-H.; Choi, J-M.; Yi, H.; Cho, S-M.; Ramadan, A.; Jeong, J-H.; Shim, J-H.; Shin, H-Ch. (2018). Development and validation of modified QuEChERS method coupled with LC–MS/MS for simultaneous determination of cymiazole, fipronil, coumaphos, fluvalinate, amitraz, and its metabolite in various types of honey and royal jelly. *Journal of Chromatography B*, 1072, 60–69.

[7] Wiesta, L.; Buleté, A.; Giroud, B.; Fratta, C.; Amic, S.; Lambert, O.; Pouliquen, H.; Arnaudguilhem, C. (2011). Multi-residue analysis of 80 environmental contaminants in honeys, honeybees and pollens by one extraction procedure followed by liquid and gas chromatography coupled with mass spectrometric detection. *Journal of Chromatography A*, 1218, 5743– 5756.

[8] Niell, S.; Jesús, F.; Pérez, C.; Mendoza, Y.; Díaz, R.; Franco, R.; Cesio, V.; Heinzen, H. (2015). QuEChERS Adaptability for the Analysis of Pesticide Residues in Beehive Products Seeking the Development of an Agroecosystem Sustainability Monitor, J. Agric. Food Chem. 63, 4484−4492.
